# Development of an Empty Sella

**DOI:** 10.1210/jcemcr/luad091

**Published:** 2023-08-14

**Authors:** Stanley M Chen Cardenas, Debraj Mukherjee, Amir H Hamrahian

**Affiliations:** Endocrinology, Diabetes, and Metabolism, The Johns Hopkins University School of Medicine, Baltimore, MD 21205, USA; Neurosurgery, The Johns Hopkins University School of Medicine, Baltimore, MD 21205, USA; Endocrinology, Diabetes, and Metabolism, The Johns Hopkins University School of Medicine, Baltimore, MD 21205, USA

**Keywords:** empty sella, hyperprolactinemia, transverse sinus stenosis

## Image Legend

**Figure 1.**
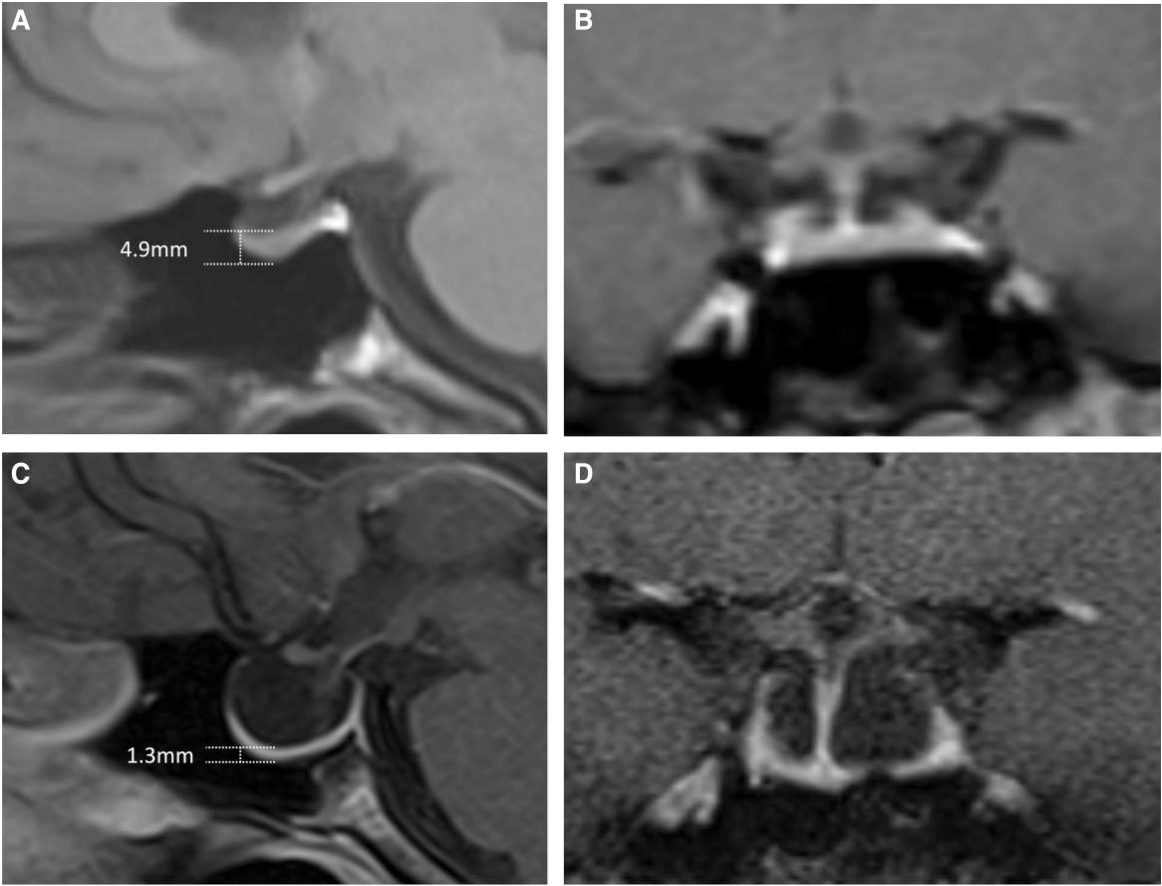
Sagittal and coronal views of pituitary gland magnetic resonance imaging at initial presentation (A-B) and after four years (C-D).

A 32-year-old woman was evaluated for hyperprolactinemia during an infertility workup. She had regular menses, no galactorrhea, and 1 successful pregnancy. The patient endorsed headaches for 6 months and blurry vision for the past few years, gradually worsening. On examination, papilledema was detected. Her prolactin level was 60.6 ng/mL (International Systems of Units: 60.6 µg/L) (4.5-23.3) with negative macroprolactinemia. Other pituitary axes were normal. Magnetic resonance imaging scans of the brain 4 years prior displayed a normal pituitary gland with minimal cupping (Fig. 1A and 1B). At that time, she did not undergo hormonal workup, and her only symptoms were blurry vision and pulsatile tinnitus. A repeat magnetic resonance imaging scan showed an empty sella (Fig. 1C and 1D). Lumbar puncture determined an elevated opening pressure of 35 cm of water (6-25), confirming increased intracranial pressure. A magnetic resonance venogram revealed a severe bilateral focal stenosis of the transverse-sigmoid sinus junction as the etiology. The patient declined stenting or ventriculoperitoneal shunt and pursued treatment with acetazolamide. Although the term “empty sella” is used, the sella is not empty. A pituitary gland height of ≤2 mm is referred to as empty sella, whereas a height of 3 to 4 mm is referred to as partial empty sella [1]. Bilateral transverse sinus stenosis commonly results in increased intracranial pressure and could lead to empty sella [2].
